# Application of Glutamine-enriched nutrition therapy in childhood acute lymphoblastic leukemia

**DOI:** 10.1186/s12937-016-0187-4

**Published:** 2016-07-11

**Authors:** Yueqin Han, Fengzhi Zhang, Jinshen Wang, Yanping Zhu, Jianhua Dai, Yueqing Bu, Qiaozhi Yang, Yingying Xiao, Xiaojing Sun

**Affiliations:** 1 Department of Pediatrics, Liaocheng People’s Hospital, Liaocheng, Shandong Province Zip 252000 People’s Republic of China; 2 Department of Nutrition, Liaocheng People’s Hospital, Liaocheng, Shandong Province Zip 252000 People’s Republic of China; 3 Department of Pediatric Hematology, Liaocheng People’s Hospital, Research direction: childhood leukemia and cancer, Liaocheng, People’s Republic of China; 4 Department of Nutrition, Liaocheng People’s Hospital, Research direction: nutrition interventions in patients with cancer and wasting syndrome, Liaocheng, People’s Republic of China

**Keywords:** Childhood acute lymphoblastic leukemia (ALL), Glutamine (Gln), Nutrition therapy, Immune function, Retinol binding protein (RBP), Urinary hydroxyproline index (UHI), Creatinine-height index (CHI), Serum albumin (ALB), Prealbumin (PA)

## Abstract

**Background:**

We investigated the effects of glutamine (Gln)-enriched nutritional therapy during chemotherapy on the nutritional status and immune function of children with acute lymphoblastic leukemia (ALL).

**Methods:**

We enrolled 48 children who were newly diagnosed with ALL in our department during the period of 2013.1-2014.12. The patients (follow random number table) were randomly divided into the control group (peptamen) and the treatment group (peptamen + glutamine), 24 cases in each group. The remission induction regimens were all based on VDLP (D) chemotherapy (VCR (Vincrisstine), DNR (Daunomycin), L-ASP (L-Asparagiase), Prednisolone and Dexamethasone). The treatment group received Gln-enriched nutritional therapy every day during the full course of chemotherapy,and the control group is as same as the treatment group except without glutamine. The indicators of general nutritional status, such as weight, height, and triceps skinfold thickness, and the indicators of biochemical tests, such as serum albumin, prealbumin, creatinine-height index, retinol binding protein, and urinary hydroxyproline index, were compared between the two groups at the end of the first, second, third and the fourth week when the chemotherapy was completed. And in the fourth week, flow cytometry was applied to detect the levels of T cell subsets and the activities of natural killer (NK) cells in peripheral blood of the two groups.

**Results:**

1. after 4 weeks nutritional therapy, there is no significant difference (*p* > 0.05) between the two groups of children in weight, height and other indicators. 2. At the end of 2 weeks treatment, the level of prealbumin (PA) and retinol-binding protein (RBP) is higher in treatment group than that in the control group (*P* <0.05), at the end of 3 weeks treatment, the thickness of triceps skinfold is higher (*P* <0.05) than that in the control group; 3. At the end of 3 and 4 weeks, the concentrations serum ALB, PA, RBP and UHI were higher than in the control group (*P* <0.05); 4. There is statistically significant (*p* < 0.05) between the two groups in edema incidence; 5. At the end of treatment (4 weeks), the percentages of CD3 +, CD4 +, CD4 +/CD8 +, NK cell are significantly decreased in the two groups (*P* <0.05).

**Conclusion:**

Gln-enriched nutritional therapy can effectively improve the systemic nutritional status of children with leukemia, improve immune function.

Acute lymphoblastic leukemia (ALL) is the most common childhood cancer, and has serious impact on children’s health. With the rapid development of molecular biology and optimization of chemotherapy, the current remission and disease-free survival rates of childhood ALL have been significantly improved, and the 5-year disease-free survival rate has reached about 90 % [1]. ALL has become an excellent example that benefits from modern cancer treatment. Intensive chemotherapy brings positive effects, but also significantly increases the incidences of chemotherapy-related complications and malnutrition, seriously affecting the quality of life of children [2–4]. Therefore, individualized treatment strategies that aim at reducing side effects related to chemotherapy and improving quality of life in children with leukemia have gained increasing attention [5, 6]. Currently, there are nutrition guidelines for adult cancer patients in China [7]. Studies in China and abroad have shown that glutamine (Gln)-containing nutritional therapy can improve the nutritional status of adult cancer patients [8, 9]. At present, nutrition therapy guidelines for children with leukemia are still missing, and there have been limited numbers of studies in this field. Clinicians in hematology generally focus on the improvement of chemotherapy, and the relapse tendency of pediatric patients with ALL. In contrast, evaluation of the overall nutritional status of children with ALL before and after chemotherapy, and the impact of nutritional status on immune function have been rarely investigated. This study aimed to explore the effects of Gln-enriched nutrition therapy that was applied during chemotherapy on the nutritional status and immune function of children with ALL, in order to investigate the mode of nutrition therapy for childhood leukemia, increase the tolerance of chemotherapy in children, enhance immunity, improve the quality of life of children, and even help to improve the long-term prognosis. When newly diagnosed with ALL, pediatric patients often have heavy tumor burden and poor general conditions. In addition, the remission induction VDLP (D) program has long treatment course and high intensity. Most children have varying degrees of edema, protein malnutrition, severe infections, and fungal infections after remission induction chemotherapy, and the incidence of serious complications after chemotherapy is high. Therefore, we choose time points before and after the VDLP (D) remission induction program in newly diagnosed ALL as the starting points of our investigations.

## Case features

Diagnosis criteria: All patients were referring to MICM classification in China “2014 Diagnosis and treatment of childhood acute lymphoblastic leukemia (ALL) (fourth Revised Draft)” [10], and were diagnosed as childhood acute lymphoblastic leukemia.

Selection criteria: the 31 new ALL patients in our hospital registered rom 2013.1.1 to 2014.12 31; with medium risk (MR) and standard risk (LR) age from 1 to 12; no CNS or testicular leukemia extramedullary infiltration symptoms; no gender and ethnic limitation; guardians of all the children voluntarily joined the treatment.

Exclusion criteria: patients with severe malnutrition, endocrine disease, such as the coexistence of severe malnutrition or diabetes at admission; coexistence of central nervous system leukemia (CNSL) or testicular leukemia; relapse children ALL; myeloid leukemia; ALL patient treated by chemotherapy in other hospital.

Grouping method: using randomized number table to group randomly;

Follow-up: 2 months follow-up after treatment to observe if there are any long-term adverse effects;

Emergency measures: when patients show serious infections, if it may relate to chemotherapy, the chemotherapy must be stopped and strengthen symptomatic and supportive treatment; if it may relate to nutritional therapy, nutritional therapy discontinue. When patients show diarrhea, vomiting and other symptoms, intravenous or oral electrolyte liquid should be given, and close monitoring the changes of electrolytes, dehydration and acid-base balance. When the nutritional therapy or chemotherapy was stopped for more than 1 week, then the patient must be excluded.

### General information

The 48 cases of newly diagnosed ALL were hospitalized in our hospital during the period of 2013.1-2014.12. There were 28 males and 20 females that were aged 14 months-11 years and 4 months (136 months), with a median age of 5 years and 6 months (66 months). The patients were randomly divided into two groups: the treatment group (peptamen + glutamine) and the control group (small peptiedes). There were no significant differences in age, sex, and severity of disease between the two groups, total of 48 children received the whole treatment, no exit and death case. The remission induction program was VDLP (D) chemotherapy. VDLP (D) method follows < 2014 Diagnosis and treatment of children with acute lymphoblastic leukemia (fourth Revised Draft) > [10]: vincristin, VCR 1.5 mg/m^2^,D_8_,D_15_,D_22_,D_29_, LR-ALL only D_8_,D_15_, daunomycin, DNR 20-30 mg/m^2^,D_8_,D_15_,D_22_,D_29_, LR-ALL only D_8_,D_15_, L-Asparaginase, L-ASP 5000-10000U/m^2^,q2d × 6-8 doses or Pegaspargase, PEG-ASP 2500U/m^2^, D_10_,D_24_, prednisolone, Pred 60 mg/m^2^, D_1-7_, dexamethasone, Dex 6-8 mg/m2, D_8-29_; All parents of children signed the informed consent form. The nutrition program was formulated by specialists in the nutrition department of our hospital and ensured that the two groups of subjects received similar amounts of calories.

### Nutritional therapy

According to WHO [11] recommended energy standard, energy requirements for all participants were calculated, in which protein 12 % to 14 %, and other nutrient intake volume refer to the recommended amount of Chinese Nutrition Society (RNI). According to the physiological requirements of each individual, nutritionists designed recipes for the patients in the two groups, and independent eating, eating habits were monitored and recorded by the nurse on duty;

Enteral nutrition method:

- Control group (independent eating + peptamen): when patients eat less than 60 % of normal physiological requirements, peptamen can provide the supplement for energy requirement (note 1), either by oral or nasal.
- Treatment group (independent eating + peptamen + glutamine): chemotherapy and glutamine during the whole treatment (note 2), when the patients eat less than 60 % of the normal physiological requirements, peptamen can provide the supplement for energy requirement (note 1), either by oral or nasal.
- Parenteral nutrition program: Different parenteral nutrition (PN) was given to those patients who cannot tolerate enteral nutrition.
- Control group: Parenteral nutrition (PN1) was given (note 3).
- Treatment group: Parenteral nutrition (PN2) (PN1 + glutamine, note 4) was given.

Treatment group:

- Note1. receipt of peptamen: Nestle products, short peptide enteral nutrition formulations, calorie 466 kcal/100 g, protein 13.7 g/100 g, the protein source is whey protein hydrolyzate from the peptide; fat: 17.5 g/100 g (soybean oil + 60 % medium-chain fatty acid + soy lecithin), carbohydrate content: 62.8 g/100 g (maltodextrin and sucrose, lactose-free).
- Note 2. receipt of glutamine: 0.4 g/kg.d, Dissolved in 100 ml of warm water or porridge, Bangshidi biotechnology companies;
- Note 3. receipt of PN1:20 % fat emulsion, amino acid injection, water-soluble vitamins, fat-soluble vitamins, Huarui pharmaceutical company;
- Note 4. receipt of PN2:PN1 + glutamine (20 % power peptide, L- alanyl -L- glutamine, 0.4 g/kg.d), Huarui pharmaceutical company.

Nutrition treatment period is 4 weeks.

### Observation indicators

#### Nutritional indicators

General nutritional indicators: The following general indicators of nutritional status were measured at the end of the first, second and third week of chemotherapy and at the fourth week after the end of chemotherapy: weight, height, and triceps skinfold thickness;

Biochemical indicators: At the above time points, fasting blood specimens were collected from the two groups. We measured the levels of serum albumin (ALB), prealbumin (PA), and retinol binding protein (RBP). Urine specimens from the same time period were collected for the detection of urine creatinine and urinary hydroxyproline proline in order to calculate the urinary creatinine-height index (CHI) and urinary hydroxyproline index (UHI). The normal reference values of serum RBP, PA, ALB were 30 ~ 70 mg/L, 200 ~ 400 mg/L, and 35 ~ 55 g/L, respectively. At the same time points, 24-h urine was collected, and urinary creatinine levels were determined by Folin colorimetry. We calculated the CHI values [24-h urine creatinine excretion (mg) of the subject/24-h urinary creatinine excretion (mg) of healthy control of the same height]. Colorimetry was applied to determine the 24-h urinary hydroxyproline excretion to calculate the UHI [urinary hydroxyproline (μmol/ml) × weight (kg)/urine creatinine (μmol/ml)].

#### Immune indicators

T lymphocyte subsets levels were recorded for the two groups of children before chemotherapy. At the end of the fourth week, we used immunofluorescence and flow cytometry (FCM) to detect the levels of T-cell subsets in peripheral blood and calculate the percentage of NK cells.

#### Edema observation

Edema indexing standard is from “Zhu Fu Tang Practical Pediatrics” eighth edition [12]:

- Mild edema: edema occurs only in the eyelid, the soft tissue of lower eye frame and, the soft tissues of tibialis anterior and ankle, after shiatsu, tissue showed mild depression, which can be quickly recovered;
- Moderate edema: visible edema in all body loose tissues, after shiatsu, tissues showed deep depression, recovery slower;
- Severe edema: visible edema in all body tissues, downcast tissues showed shiny tension, even with liquid exudation; it may also be associated with effusion in chest, abdomen and sheath.

### Statistical analysis

Statistical analysis was conducted using the SPSS 14.0 software. The measurement data were expressed as mean ± standard deviation (x ± s), intergroup comparison was conducted using the *t* test. Repeat sequence data using analysis of variance and chi-square test was used for the comparison of rate. *P* <0.05 was considered statistically significant.

## Results

1. Before treatment, there is no significant difference (*P* > 0.05) between the two groups in weight, height, and triceps skinfold thickness;
2. after 4 weeks nutrition therapy, there is no significant difference (*P* > 0.05) in height and weight of the children in the two groups, compared with that before the treatment;
3. after the 3 weeks treatment, the triceps skinfold thicknesses in the two groups are higher than those in the control group (*P* <0.05) (Table 1).

**Table 1:** General nutritional indicator of the two groups of ALL Children at the end of the 1st、2nd、3rd and the 4th week (x ± s)

Parameters	*n*	Weight (kg)	Height (cm)	Triceps skinfold thickness (mm)
Treatment group	24			
Before treatment		29.02 ± 1.23	116.71 ± 4.26	12.09 ± 1.69
The end of 1st week		28.98 ± 3.00	116.74 ± 4.33	12.12 ± 1.87
The end of 2nd week		27.46 ± 2.53	116.76 ± 4.36	11.45 ± 1.79
The end of 3rd week		29.65 ± 3.24	116.79 ± 4.34	12.62 ± 1.86
The end of 4th week		30.78 ± 3.76	116.80 ± 4.32	13.45 ± 1.73
Control group	24			
Before treatment		29.28 ± 2.67	117.24 ± 3.68	12.02 ± 1.09
The end of 1st week		29.21 ± 2.83	117.26 ± 3.99	11.97 ± 1.21
The end of 2nd week		28.57 ± 2.14	117.29 ± 3.95	10.78 ± 1.15
The end of 3rd week		30.02 ± 3.51	117.31 ± 3.86	10.34 ± 1.24^a^
The end of 4th week		29.53 ± 3.21	117.32 ± 3.82	11.12 ± 1.12

^a^compare with control group, *P* < 0.05

### Before treatment, there is no statistically difference (*P* > 0.05) in ALB, PA, RBP, CHI ad UHI for the two groups

1. At the end of first week, there is no statistically difference (*P* > 0.05) in ALB, PA, RBP, CHI and UHI for the two groups;
2. At the end of second week, the level of PA and RBP in treatment group is higher than that in the control group (*P* < 0.05), and there is no statistically difference in ALB, CHI and UHI;
3. At the end of third and fourth week, the levels of ALB, PA, RBP and UHI are higher in the treatment group than that in the control group (*P* < 0.05), and there is no statistically difference in CHI for the two groups (Table 2).

**Table 2:** General nutritional and biochemical indicator measurements of the two groups of ALL patients at the end of the 1st、2nd、3rd and the 4th week (x ± s)

Parameters	*n*	ALB (g/L)	PA (mg/L)	RBP (mg/L)	UHI	CHI
Treatment group	24					
Before treatment		29.45 ± 2.87	106.6 ± 21.58	19.56 ± 1.78	1.38 ± 0.48	75.96 ± 11.69
At the end of 1st week		29.34 ± 3.53	107.5 ± 22.47	19.42 ± 1.94	1.39 ± 0.55	76.21 ± 12.15
At the end of 2nd week		28.69 ± 2.72	113.47 ± 20.56^a^	21.76 ± 1.89^a^	1.35 ± 0.51	72.54 ± 13.21
At the end of 3rd week		30.78 ± 2.75^a^	130.52 ± 22.31^a^	22.34 ± 2.15^a^	1.42 ± 0.55^a^	75.88 ± 14.16
At the end of 4th week		32.57 ± 3.05^a^	145.71 ± 23.25^a^	24.59 ± 5.30^a^	1.49 ± 0.57^a^	78.22 ± 14.57
Control group	24					
Before treatment		29.34 ± 3.67	111.78 ± 21.23	20.57 ± 2.32	1.29 ± 0.51	75.79 ± 12.33
At the end of 1st week		29.11 ± 3.42	110.44 ± 20.54	20.13 ± 2.02	1.28 ± 0.46	75.59 ± 12.08
At the end of 2nd week		28.77 ± 3.09	100.85 ± 19.78	19.78 ± 2.11	1.26 ± 0.41	73.14 ± 12.79
At the end of 3rd week		26.90 ± 3.54	112.62 ± 20.01	20.05 ± 2.52	1.15 ± 0.38	72.21 ± 12.58
At the end of 4th week		27.15 ± 3.29	110.57 ± 21.22	19.52 ± 2.49	1.18 ± 0.36	72.75 ± 12.93

^a^compared with control group, *P* < 0.05

### At the end of fourth week, edema incidence is 20.83 % in the treatment group and 50 % in the control group (*X*^2^ = 4.46, *P* < 0.05), there is no statistically difference. There are more severe edema cases in the control group than that in the treatment group (*X*^2^ = 0.437,*P* > 0.05) (Table 3)

**Table 3:** Edema after 4 weeks treatment

Parameters	*n*	Cases of edema	Cases of severe edema	Percentage of edema (%)
Treatment group	24			
		5	1	20.83 %^a^
Control group	24			
		12	4	50 %

^a^compared with control group,*P* < 0.05

1. Before treatment, there is no statistically difference in the percentage of CD3+, CD4+, CD4+/CD8+ and NK cells for the two groups (*P* > 0.05);
2. After 4 weeks chemotherapy, there is statistically difference in the percentage of CD3+, CD4+, CD4+/CD8+ and NK cells (*P* < 0.05) for the control group, and there is statistically difference in CD3+, CD8+ and NK cells (*P* < 0.05) for the treatment group;
3. After 4 weeks chemotherapy, there is statistically difference in CD3+, CD4+, CD4+/CD8+ and NK cells for the control and treatment groups (*P* < 0.05) (Table 4).

**Table 4:** the differences of cellular immune function in the two groups before and after treatment

Parameters	Control group (*n* = 24)		Treatment group (*n* = 24)	
	Before chemotherapy	after 4 weeks	Before chemotherapy	after 4 weeks
CD3+	69.23 ± 1.15	55.20 ± 2.96^a^	70.55 ± 3.05^a^	63.67 ± 1.64^b^
CD4+	32.74 ± 2.85	26.30 ± 2.70^a^	33.94 ± 2.75	33.83 ± 1.56^b^
CD8+	28.45 ± 0.98	28.00 ± 0.88	27.79 ± 0.89^a^	26.50 ± 1.02^b^
CD4+/CD8+	1.16 ± 0.58	0.93 ± 0.10^a^	1.22 ± 0.65	1.28 ± 0.84^b^
NK cell %	23.95 ± 2.34	16.23 ± 4.15^a^	25.47 ± 2.01^a^	19.54 ± 4.06^b^

^a^compared with before chemotherapy,*P* < 0.05

^b^compared with control group,*P* < 0.05

## Discussions

The complete remission rate and disease-free survival rate of childhood ALL have been significantly improved. This improvement can be attributed to accurate MICM classification and correct assessment of disease severity of pediatric patients. But it is mainly due to the application of L-asparaginase (L-ASP) and other chemotherapy drugs. L-ASP, as a potent anticancer drug, is widely used in childhood ALL chemotherapy, and plays a milestone role in combination chemotherapy [13–15]. The anti-tumor mechanism of L-ASP has not been elucidated, but it has been widely accepted that the potential target for its role is to inhibit protein synthesis [16]. At the same time that L-ASP exerts the effective anti-tumor effect, it also produces a series of adverse reactions such as myelosuppression, hypersensitivity, blood clotting abnormalities, and gastrointestinal symptoms, making L-ASP a double-edged sword in the treatment of childhood ALL. Children with ALL are in conditions that catabolism is in the dominant state. Patients have a serious shortage of calories and protein intake, and have an increased consumption of body protein, while liver infiltration and liver damage can further affect protein synthesis. Application of L-ASP-containing chemotherapy further inhibits protein synthesis, eventually leading to protein malnutrition, decreased immune function, increased mortality, and seriously affecting the subsequent chemotherapy. Owens JL [17] suggested that declining nutritional status of children with leukemia after chemotherapy is a potential risk factor, and it can reduce immune function, change drug metabolism, enhance drug toxicity, and prolong wound healing. There are currently no uniform guidelines on nutrition therapy of children with cancer and no optimal nutrition formula targeting nutrition deficiency in children with leukemia due to chemotherapy. It is thus beneficial to establish unified diagnostic criteria for malnutrition and evidence-based nutrition guidelines for children with acute lymphocytic leukemia for appropriate nutritional intervention. In 2009, the American Society of Parenteral and Enteral Nutrition (ASPEN) published clinical nutritional guidelines for cancer patients [18], emphasizing that there is no evidence to indicate that nutrition therapy can promote the growth of tumour cells. It provides a theoretical basis for us to apply nutrition therapy during the chemotherapy for children with ALL. Based on our successful experience in nutrition therapy for adult cancer patients, we used enhanced Gln nutrition therapy in pediatric ALL patients during chemotherapy, and observed the effects on the immune function and nutrition indicators of children.

Gln is the most abundant non-essential amino acid in the muscle, accounting about 60 % of total free amino acids in the human body. It has been proven that Gln has multifaceted roles. For example, Gaurav K et al. [19] found that Gln could reduce mucosal injury caused by radiotherapy and chemotherapy in patients with acute leukemia, as well as gastrointestinal toxicity caused by 5-FU, reduce the paclitaxel-related myalgia and arthralgia, prevent paclitaxel neurotoxicity, alleviate the immunocompromised state of laboratory animals after MTX chemotherapy, and stimulate the recovery of neutrophils in patients with myeloid leukemia after chemotherapy. Sornsuvit C et al. [20] also confirmed that parenteral supplementation of glutamine dipeptide could improve neutrophil function in patients with myeloid leukemia, reduce chemotherapy-related oral mucositis, and improve the nutritional status of patients.

The specific mechanism of Gln is not fully understood. Clinical and basic research has been conducted to reveal the mechanisms underlying Gln functions. Goto M et al. [21] revealed in the study of acute myeloid leukemia (AML) that Gln might play an important role in energy metabolism in the Krebs cycle through its effects on redox balancing. Emadi A et al. [22] found that the selective inhibition effects of Gln led to the growth inhibition of myeloid leukemia cells with defects in the IDH gene. Based on the Warburg effect theory, there have been trials using chemical metabolic inhibitors to treat hematological malignancies, including childhood ALL [23, 24]. The above studies provide a theoretical basis for the application of Gln in the adjuvant treatment of leukemia. But there are only limited numbers of reports using Gln as an immune and nutrition agent and analyzing the effects of Gln on the nutritional status and immune function of children with ALL from the point of view. Only a couple of studies conducted small-sample short-course observations [25], and there is still the lack of large-sample, and multi-center in-depth studies.

Evaluation of nutritional condition indicators in children with leukemia after chemotherapy is extremely complex. The standard parameters used to assess the nutritional status of children with cancer may vary very often. For example, edema after steroid treatment can mask malnutrition (weight gain leading to fluid retention), thus impairing the accuracy of using weight as an indicator for nutritional assessment. Serum protein markers can show bias if used for the evaluation of malnutrition caused by infections and sepsis. Retinol-binding protein is the most sensitive indicator reflecting protein nutrients in diet. In the presence of normal renal function, CHI is a reliable biochemical parameter measuring muscle protein consumption. Creatinine is a creatine metabolite, and its excretion is closely related to total muscle content, body surface area and weight. Creatinine is not affected by infusion and fluid retention and is more sensitive than indicators such as nitrogen balance and serum ALB. Hydroxyproline is a product of collagen metabolism. Children with malnutrition and those with reduced protein levels in the body have reduced urinary excretion of hydroxyproline. Therefore, UHI can be used as a reliable biochemical indicator to determine the protein nutrition status of children. Flow cytometry was used to detect the indicators for immune function in children, and detection of CD3+, CD4+ CD8+, and NK cell numbers is the classic means to assess immune function. These indicators are sensitive, practical, stable and reproducible, and are suitable for assessing the nutritional status and immune function of pediatric patients. Therefore, we selected these indicators to assess changes in the nutritional status and immune function of our patients. It was found that after the first week of chemotherapy, the two groups of children did not show significant differences in weight, ALB, PA, CHI, RNP, and UHI (*p* > 0.05), after the second week of chemotherapy, the pediatric patients in the control group showed varying degrees of reduction in weight and triceps skinfold thickness and slow decline in ALB, PA, RNP, CHI, and UHI, and after the third week of chemotherapy, both groups had increased weight (due to the effect of glucocorticoids), whereas the control group showed an increased proportion of children with varying degrees of edema, and significant decreases in ALB, PA, RNP, CHI, and UHI, with significant differences as compared to the treatment group (*p* <0.05). Children in the control group had more cases with severe edema, and the ALB, PA, RNP, CHI, and UHI levels were significantly lower than those of the treatment group, with significant differences between the two groups (*p* <0.05). There was no significant difference in height between the two groups.

NK cells are the main effector cells in immune cells, and detecting the level of NK cells is important to assess the disease initiation, evolution and prognosis of outcome. So the cellular immune function in the two groups was tested before and after 4 weeks chemotherapy [26, 27]. Glutamine plays an important role in immune regulation, which is required for secretion, proliferation and function maintenance of lymphocyte. As precursors and primary energy for nucleic acid biosynthetic, glutamine can promote mitosis, differentiation and proliferation of lymphocytes and macrophages, and increase the generation of TNF cytokine and IL-1 and the mRNA synthesis of phospholipids. Providing exogenous glutamine can significantly increase the total number of lymphocytes and the ratio of CD4+/CD8+ in T lymphocytes and circulation, and enhance immune function in critically ill patients, [28–30]. In this study, glutamine was applied to the leukemia patients receiving chemotherapy, similar results were also observed.

Our research data showed that after 4 weeks chemotherapy, the percentages of CD3 +, CD4 +, CD4 +/CD8 +, and NK cell in the two groups were significantly decreased and there were statistically significant differences (*P* <0.01) as compared with that before treatment, which indicated that the cellular immune function in both groups was seriously damaged, which suggested that strengthened glutamine therapy can promote the recovery of immune function in ALL patients.

In this study, we found that Gln-enhanced nutrition therapy can significantly improve the nutritional status of children with ALL, and it can also improve the body’s metabolism and nitrogen balance, promote protein synthesis, and increase the total number of lymphocytes. Regarding the safety of glutamine application, in a chronic toxicity test Wong AW et al. [31] found that after given the rats of Gln at the dose of 3832-4515 mg/kg for 13 weeks, there were no obvious toxic reactions found.

During the treatment, all pediatric patients did not show adverse reactions, including allergic reactions, diarrhea, bloating, vomiting, and constipation, and most patients showed good tolerance. This therapy is economical, practical and worthy of promotion in clinical practices.

The essential amino acids, such as glutamine, can be synthesized in human body. However, at the beginning of the experiment, the concentrations of glutamine of the two groups children were not obtained, so the impacts of the amount of glutamine synthesized by human body on the experiment results cannot be received, which is deficiency of the experimental design. In the future experiments, we will take care of that, and make our results more robust.

## Conclusions

Gln-enriched nutritional therapy can effectively improve the systemic nutritional status of children with leukemia, improve immune function.
